# Matrix Metalloproteinase-1 and -9 in Human Placenta during Spontaneous Vaginal Delivery and Caesarean Sectioning in Preterm Pregnancy

**DOI:** 10.1371/journal.pone.0029855

**Published:** 2012-01-12

**Authors:** Deepali P. Sundrani, Preeti M. Chavan-Gautam, Hemlata R. Pisal, Savita S. Mehendale, Sadhana R. Joshi

**Affiliations:** 1 Department of Nutritional Medicine, Interactive Research School for Health Affairs, Bharati Vidyapeeth University, Pune, India; 2 Department of Obstetrics and Gynecology, Bharati Medical College and Hospital, Bharati Vidyapeeth University, Pune, India; Max Delbrueck Center for Molecular Medicine, Germany

## Abstract

Preterm birth is a major public health problem in terms of loss of life, long-term and short term disabilities worldwide. The process of parturition (both term and preterm) involves intensive remodelling of the extracellular matrix (ECM) in the placenta and fetal membranes by matrix metalloproteinases (MMPs). Our previous studies show reduced docosahexaenoic acid (DHA) in women delivering preterm. Further omega 3 fatty acids are reported to regulate MMP levels. This study was undertaken to examine the placental levels of MMPs and their association with placental DHA levels in women delivering preterm. The levels of MMP-1 and MMP-9 in 74 women delivering preterm (52 by spontaneous vaginal delivery and 22 by caesarean sectioning) and 75 women delivering at term (59 by spontaneous vaginal delivery and 16 by caesarean sectioning) were determined by enzyme-linked immunosorbent assay (ELISA) and their association with placental DHA was studied. Placental MMP-1 levels were higher (p<0.05) in women delivering preterm (both by spontaneous vaginal delivery and caesarean sectioning) as compared to those delivering at term. In contrast, placental MMP-9 levels in preterm pregnancies was higher (p<0.05) in women with spontaneous vaginal delivery while lower (p<0.05) in women delivering by caesarean sectioning. Low placental DHA was associated with higher placental MMP-9 levels. Our study suggests a differential effect of mode of delivery on the levels of MMPs from placenta. Further this study suggests a negative association of DHA and the levels of MMP-9 in human placenta although the mechanisms need further study.

## Introduction

Preterm birth, defined as birth before 37 weeks of gestation accounts for 12% of all births worldwide [1]. It is a major determinant of neonatal mortality and morbidity and has long-term adverse consequences for health [2]. Preterm birth is usually preceded by preterm premature rupture of the fetal membranes, which occurs when the strength of the membranes is compromised and is defined as rupture before 37 completed weeks of gestation [3]. During pregnancy, matrix metalloproteinases (MMPs) are reported to contribute in extracellular matrix (ECM) remodelling/degradation leading to cervical ripening, fetal membrane rupture and finally placental separation from maternal uterus [4]–[6].

MMPs are a family of homologous zinc-dependent endopeptidases that are classified based on their structure and function as collagenases, gelatinases, stromelysins, matrilysins and membrane type MMPs (MT-MMPs) [7], [8]. MMP-1 is a member of the collagenase subgroup, that degrade fibrillar collagen types I, II and III while MMP-9 is a member of gelatinase subgroup, which mainly digests peptide bonds in denatured collagens (gelatins) to yield small peptides [9]. MMPs and tissue inhibitors of metalloproteinases (TIMPs) are also suggested to play a crucial role in normal placental functions [10].

Most studies have reported changes in MMP-1 and MMP-9 in serum, aminiotic fluid and fetal membranes during preterm pregnancy [5], [11]–[15]. However, since the detachment of the placenta requires ECM degradation in which MMPs are involved there is a need to examine the levels of MMPs in the placenta for better understanding of their role in preterm delivery. Studies have examined the gene expression of MMP-9 from the placenta in preeclampsia and term deliveries but not in preterm placenta [16]. Further, to our knowledge there are no studies which have examined the protein levels of MMP-1 and MMP-9 in preterm placenta with respect to the mode of delivery i.e. spontaneous vaginal delivery or caesarean sectioning.

The activity of MMPs like MMP-2, -3, and -9 are reported to be regulated by long chain polyunsaturated fatty acids (LCPUFA) [17]–[19]. Our earlier studies in preterm deliveries indicate increased oxidative stress and reduced LCPUFA especially docosahexaenoic acid (DHA) levels in preterm pregnancy [20], [21].

**Figure 1:**
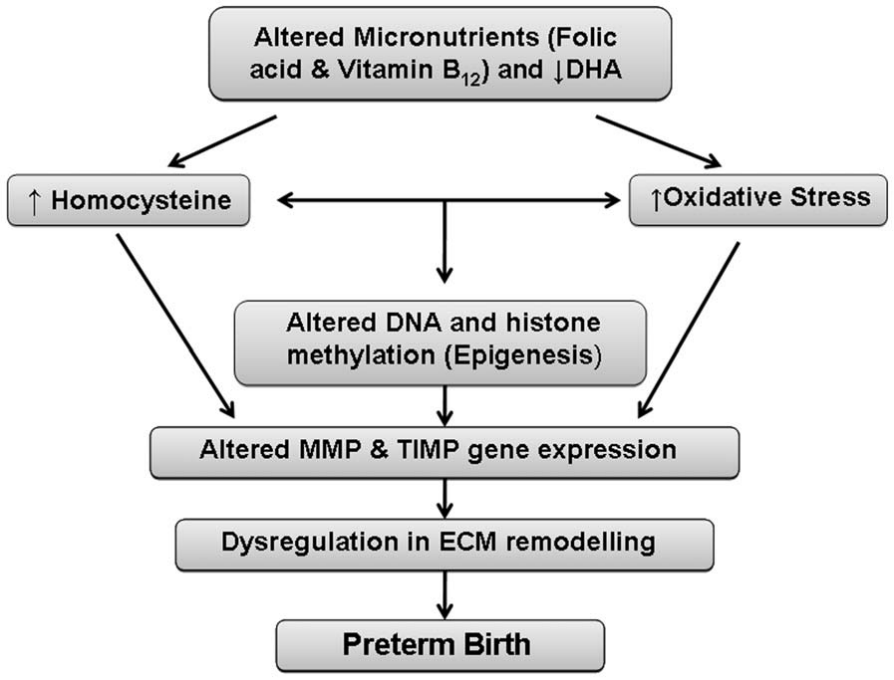
Role of altered micronutrients and DHA in the epigenetic regulation of MMP and TIMP genes leading to preterm birth.

Based on all our findings we have recently hypothesized that altered intake or metabolism of maternal vital micronutrients (folic acid, vitamin B_12_) and omega 3 fatty acids especially DHA influences the one carbon cycle thereby contributing to increased homocysteine and oxidative stress leading to altered epigenetic regulation of MMP and TIMP gene expression in women delivering preterm babies [22] (Figure 1). This study was undertaken to examine the levels of MMP-1 and MMP-9 in preterm placenta and their association with placental DHA levels.

## Methods

### Subjects

This study was conducted at the Department of Obstetrics and Gynaecology, Bharati Hospital, Pune during the year 2008–2010. This study was conducted with the understanding and consent of each subject and was approved by the Bharati Vidyapeeth Medical College Institutional Ethical Committee. A total number of 149 pregnant women with singleton pregnancy were recruited for this study. 75 women delivered at term, 59 (78.66%) by spontaneous vaginal delivery and 16 (21.33%) by caesarean sectioning. 74 women delivered preterm, 52 (70.27%) by spontaneous vaginal delivery and 22 (29.72%) by caesarean sectioning. The preterm (PT) group consisted of women delivering preterm (total gestation <37 weeks) without complications such as pre-eclampsia, gestational diabetes and anemia. Preterm cases with chorioamnionitis and fetal infections were excluded from the study since it is well recognized that infections can cause premature labor. The control group consisted of pregnant women delivering at term (total gestation ≥37 weeks) and having no medical or obstetrical complications.

Women were excluded from the study if there was evidence of other pregnancy complications, such as multiple gestation, chronic hypertension, type I or type II diabetes mellitus, seizure disorder and renal or liver disease. Pregnant women with alcohol or drug abuse were also excluded from the study. All women were routinely given iron tablets as per the National Prophylaxis programme. Gestational age was calculated by last menstrual period and then confirmed by ultrasound. All recruited women were from the lower socioeconomic group and well matched for dietary and lifestyle patterns.

### Tissue collection and processing

Placental tissues: Fresh placental tissues were obtained from normal and preterm pregnancies immediately after delivery. Fetal membranes were trimmed off and the placenta was weighed. Small pieces (approximately 1 cm (l)×1 cm (w)×0.5–1 cm (h)) were cut out from five different regions of the placental cotyledon. The tissue pieces were individually rinsed in phosphate buffer saline (PBS) to wash off maternal and fetal blood. Tissue pieces were collected in liquid nitrogen and stored at −80°C until assayed.

### Preparation of tissue lysates and estimation of total protein

100 mg of placental tissue was weighed and centrifuged twice with 1× PBS at 4°C. The supernatant was discarded and pellet was collected. The tissue pellet was lysed in chilled cell lysis buffer (50 mM TRIS HCl, 150 mM NaCl, 1 mM EDTA, 1 mM phenyl methane sulfonyl fluoride (PMSF), 10 µM Leupeptin, 0.1 µM Aprotinin) for 30 mins on ice with intermittent vortexing. The extract was then centrifuged at 28341.30 g for 10 mins at 4°C. The clear supernatant (lysate) was then transferred to clean tube. Total protein content of the lysates was estimated by Lowry method [23].

### MMP-1 and MMP-9 levels from placental tissue lysates

MMP-1 and MMP-9 levels were measured from placental tissue lysates using commercial enzyme-linked immunosorbent assay (ELISA) kits (RayBiotech, Norcross, GA, U.S. for MMP-1 and Abnova, Taipei, Taiwan for MMP-9). The detection limit (sensitivity) of the assay was 8 pg/ml for MMP-1 and 50 pg/ml for MMP-9. All assays were carried out by personnel who were blinded to the study. MMP-1 and MMP-9 concentrations have been normalized for 1 mg of total protein content.

### Statistical analysis

Values are mean ± SD. The data were analyzed using SPSS/PC+ package (Version 11.0, Chicago IL, USA). Mean values of the various parameters were compared using unadjusted independent sample t-tests to identify statistically significant differences (p<0.05). Skewed variables were transformed to normality using the following transformations: log to the base 10 (MMP-1, MMP-9, and DHA). Correlation between variables was studied using Pearson's correlation analysis after adjusting for gestation, age and body mass index (BMI).

## Results

### Maternal characteristics and birth outcome

The maternal characteristics and birth outcomes are given in Table 1. All the women recruited in the study had similar age, income, education and parity. The body mass index (BMI) and gestation of women delivering preterm were significantly lower (p<0.01) than that of women delivering at term. The placenta weight of women delivering preterm was significantly lower (p<0.01) than that of women delivering at term. The baby weight, length, head and chest circumference were also lower (p<0.01 for all) in the preterm group as compared to term.

**Table 1:** Maternal characteristics and birth outcome.

	Term (n = 75)	Preterm (n = 74)
Maternal characteristics		
Age (yr)	23.1±3.7	22.6±4.0
Weight (kg)	51.0±8.6	46.6±8.6**
Height (cm)	151.6±4.8	150.6±5.2
Body Mass Index (kg/m^2^)	22.2±3.6	20.7±3.1**
Gestation (wks)	39.3±1.1	34.5±1.9**
Education (grade)	9.9±3.7	9.3±3.1
Income (Rs.)	5319.4±2558.2	4971.4±3686.9
Placental Wt. (gm)	511.8±84.6	414.5±94.1**
Baby wt. (kg)	2.8±0.2	2.0±0.4**
Baby length (cm)	48.2±2.9	44.9±3.4**
Baby head circumference (cm)	34.0±1.4	31.5±2.2**
Baby chest circu mference (cm)	32.4±1.5	28.8±3.1**

Values given are mean ± SD.

**p<0.01 when compared to term.

### Placental MMP-1 concentrations

**Figure 2:**
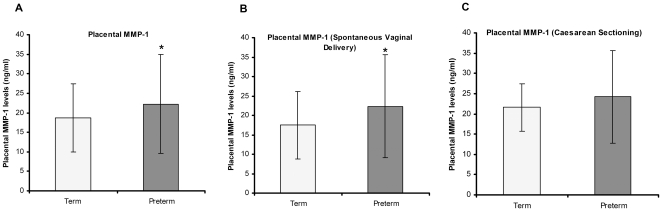
Placental MMP-1 levels. (A) Comparison of placental MMP-1 levels in preterm and term groups, *p<0.05. (B) Comparison of placental MMP-1 levels in preterm and term groups undergoing spontaneous vaginal delivery, *p<0.05. (C) Comparison of placental MMP-1 levels in preterm and term groups undergoing caesarean sectioning.

Placental MMP-1 levels were significantly increased in PT group (22.22±12.69 ng/ml) (p<0.05) as compared to control (18.64±8.7 ng/ml) (Figure 2a). We also examined the levels of MMP-1 based on the mode of delivery. Placental MMP-1 levels were significantly increased in PT group (22.35±13.29 ng/ml) (p<0.05) as compared to control (17.53±8.68 ng/ml) in women undergoing spontaneous vaginal delivery (Figure 2b). Similarly, placental MMP-1 levels were also increased but not significant in PT group (24.20±11.46 ng/ml) as compared to control (21.58±5.87 ng/ml) in women undergoing caesarean section (Figure 2c).

### Placental MMP-9 concentrations

**Figure 3:**
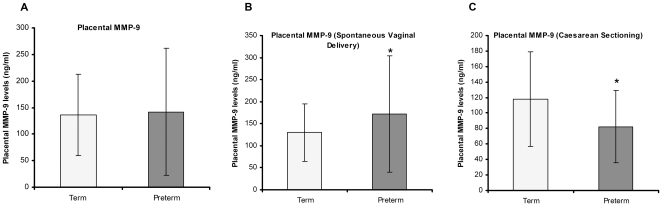
Placental MMP-9 levels. (A) Comparison of placental MMP-9 levels in preterm and term groups. (B) Comparison of placental MMP-9 levels in preterm and term groups undergoing spontaneous vaginal delivery, *p<0.05. (C) Comparison of placental MMP-9 levels in preterm and term groups undergoing caesarean sectioning, *p<0.05.

Placental MMP-9 levels were increased but not significant in PT group (141.97±119.05 ng/ml) as compared to control (136.56±76.83 ng/ml) (Figure 3a). We also examined the levels of MMP-9 based on the mode of delivery. Placental MMP-9 levels were significantly increased in PT group (172.38±131.76 ng/ml) (p<0.05) as compared to control (130.46±65.26 ng/ml) in women undergoing spontaneous vaginal delivery (Figure 3b). In contrast, placental MMP-9 levels were significantly decreased in PT group (82.53±47.11 ng/ml) (p<0.05) as compared to control (117.87±61.27 ng/ml) in women undergoing caesarean section (Figure 3c).

### Placental DHA levels

We have earlier reported reduced levels of placental LCPUFAs especially DHA in preterm deliveries. In that study, placental DHA levels were lower (2.05±0.97 gm/100 gm of fatty acids, p<0.01, n = 58) in women delivering preterm compared to those delivering at term (3.19±0.94 gm/100 gm of fatty acids, n = 44) [21].

### Associations of placental MMP-9 and DHA levels

**Figure 4:**
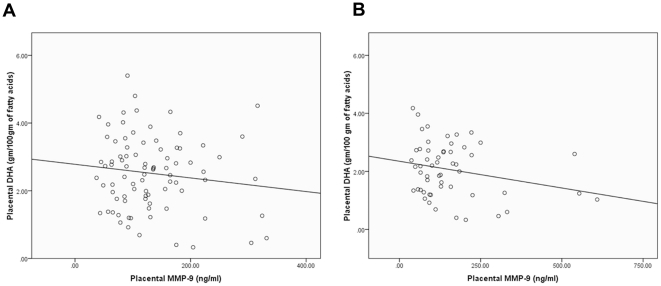
Associations between placental MMP-9 and placental DHA levels. (A) Whole Cohort, r = −0.213, p = 0.046, n = 88 (B) Preterm group, r = −0.284, p = 0.038, n = 54.

Data on both MMP and DHA levels were available on 34 term and 54 preterm women. The associations between MMP and DHA levels were studied on this subset of term (n = 34) and preterm (n = 54) placental samples for which DHA levels were estimated in our previous study [21]. A negative association between placental MMP-9 and placental DHA (r = −0.213, p = 0.046, n = 88) was seen in the whole cohort (Figure 4a). Further a negative association between placental MMP-9 and placental DHA levels (r = −0.284, p = 0.038, n = 54) was also seen in the PT group (Figure 4b). In contrast there was no association of MMP-1 with DHA in the whole cohort or individual groups.

## Discussion

The present study reveals several novel and interesting key findings in pregnant women delivering at term and women delivering preterm who were matched for age, gestation and socioeconomic status. Our results show: (1) increased placental MMP-1 levels in women delivering preterm compared to those delivering at term by both spontaneous vaginal delivery as well as caesarean sectioning; (2) increased placental MMP-9 levels in women delivering preterm compared to those delivering at term by spontaneous vaginal delivery; (3) reduced MMP-9 levels in women delivering preterm compared to those delivering at term by caesarean sectioning; (3) negative association between placental MMP-9 levels and placental DHA levels in the whole study cohort as well as in preterm group.

It is well established that the normal growth, development and function of placenta are crucial for successful pregnancy [24]. As mentioned earlier, MMPs contribute in ECM remodelling/degradation required in the processes of preterm and term human parturition. There are a number of studies examining the levels of MMP-1 and MMP-9 in the fetal membranes and amniotic fluid both in term and preterm labor indicating their role in parturition [1], [11], [13], [25], [26]. However, there is no information on the placental levels of these MMPs in preterm deliveries. In our study, placental MMP-1 levels were significantly increased in women delivering preterm compared to those delivering at term (irrespective of mode of delivery), suggesting that placental MMP-1 levels may be involved in initiation of parturition through altered extracellular remodelling of placental tissue in preterm deliveries. These results are consistent with earlier studies that show increased MMP-1 levels in amniotic fluid in preterm deliveries [13]. Increased placental expression of MMP-1 gene has been previously shown on a small sample size (n = 5) in women undergoing normal spontaneous deliveries at term compared with non-laboring caesarean deliveries at term [1]. However, there is no information on changes in placental MMP-1 levels in women delivering preterm and term by vaginal delivery or caesarean section. Our study for the first time shows increased placental MMP-1 levels in preterm group as compared to control in women undergoing spontaneous vaginal delivery as well as those undergoing caesarean section suggesting that MMP-1 has a crucial role in parturition during preterm birth.

Further our results show increased levels of MMP-9 in preterm placenta as compared to term in women undergoing spontaneous vaginal delivery. However, MMP-9 levels were lower in preterm placenta as compared to term in women undergoing caesarean section. MMP-9 has been suggested to be important in separation of the placenta from the uterine wall during labor [27]. An increase in MMP-9 expression is suggested to contribute to degradation of the ECM in the fetal membrane and placenta, thereby facilitating fetal membrane rupture and placental detachment from the maternal uterus at labor, both preterm and term [5]. Studies by Fortunato et al. have also shown that MMP-9 is increased in the amniotic fluid of women with premature rupture of membrane (PROM) [12]. Previous studies have reported increased activity of MMP-9 in fetal membrane [14], [25], [28] myometrium [29], [30] and placenta [5] at the time of labor at term. None of these studies have examined the effect of mode of delivery on placental levels of MMP-9 in term and preterm delivery. Our findings suggest that the mode of delivery may be one of the factors affecting the placental levels of MMP-9.

Our findings for the first time indicate a negative association of placental DHA with MMP-9. We have earlier reported reduced levels of omega 3 fatty acids especially DHA in preterm deliveries. It is known that supplementation with omega 3 fatty acids reduce 2-series prostaglandin (PG) production [31]. Studies have shown that prostaglandins upregulate MMP expression and activity during labor [11], [32], [33]. Studies in rat vascular smooth muscle cells have suggested that DHA significantly decreases the MMP activity [34]. It has also been shown that DHA supplementation (n-3 fatty acid intake) in rats reduces the expression of placental MMPs (MMP- 2 and 9) [35]. Downregulation of MMP-9 by omega 3 fatty acid supplementation has also been shown in humans [36]. It is likely that reduced omega 3 fatty acids in preterm deliveries, upregulate 2-series prostaglandin release thereby increasing MMP-9 levels in preterm deliveries. Further studies are needed to confirm this mechanism.

In conclusion our present study indicates increased placental levels of MMP-1 and MMP-9 in preterm deliveries. Further, for the first time we report a differential effect of mode of delivery on MMP-9 levels in the preterm placenta. The increased placental MMP-1 levels in preterm group as compared to control may indicate a crucial role of this proteinase in preterm birth. We have earlier reported the role of DHA in the one carbon cycle thereby influencing epigenetic patterns [37], [38]. We have recently hypothesized that an altered one carbon metabolism in preterm delivery may influence the epigenetic regulation of MMP and TIMP gene expression in women delivering preterm babies [22]. Studies are ongoing in our laboratory for examining the epigenetic regulation of MMPs and TIMPs in preterm delivery.
